# A Fresh Start with the Summer

**Published:** 1888-06-09

**Authors:** 


### The Doctor's Pulpit.

### A FRESH START WITH THE SUMMER.

The east wind for the present season is gone, or going. On the principle that the worst knave is not too bad to be represented by counsel, we entered a plea on its behalf some weeks ago, and showed that it was not altogether without redeeming qualities. Nevertheless, in common with all our readers, our regard for the east wind is a feeling not unmingled with fear. It is a dangerous enemy, which, like fire and water, may sometimes be a good servant, but is a very bad master. It gets the credit of being a special enemy to the lungs; and so it is. Perhaps, however, its havoc among livers is much more extensive, if not so severe, as its inroads upon lungs. A chilled liver produces a variety of distressing worries, which are often not very easily accounted for. A sudden change of the wind to the east makes us stupid, owlish, cross, and incapable, without our exactly knowing the reason why. The enemy has left the lungs alone and seized upon the liver.

Thanks, however, to the flight of time, we may congratulate ourselves upon the approach of the fair season of summer. The change is delightful, and the feeling of delight is a correct measure of the gain to the general health which the changed season has brought with it. The early summer in the British Isles is the healthiest season of the year. The busy doctor, worn with cares, and anxious cases, from November to the end of May, calls a halt in June. Some of his patients fly to the seaside, and those who cannot do so seek the shady suburban roads or the green parks, and bid adieu to physic. The doctor himself, more thankful than his patients, heaves a sigh of relief, and looks forward to three months of only moderate work. Poor doctor! He of all men can enter fully into the feelings of that ancient but overdriven charwoman who, at the age of seventy, was so glad to die because in the future world she was "going to do nothing for ever and ever."

The beginning of summer is the beginning of hope. The old man who has struggled through another winter, and escaped bronchitis and pneumonia, takes heart of grace again, and toddles about with renewed courage and fresh briskness. He feels safe and well in the genial warmth. He may serve as an index to those who are younger and stronger. Before them, as before him, there is a period of "promise and potency." The summer, if well used, should increase the store of health, and prepare all of us for the sterner struggle with climate and possible disease which awaits us in the winter.

How is the summer to be made use of so as to produce its best effects on the health? The question is large and comprehensive, and may properly be answered in some detail. The summer is, *par excellence*, the season of open-air life. Town people may not be aware of the fact, but in the country the cattle, which have been carefully housed for the night since the end of October, are turned out in the middle of May to sleep under the only roof which Nature provides—the open sky. They are not injured, but rather benefited by the sudden change to open-air sleeping accommodation. The lesson to be learnt is—not that we should all take our beds out into the garden, or throw off all our winter flannels at one swoop. *Festina lente* is the motto for spring. Those who have not slept with windows open during the winter should now open them at least an inch or two, whilst those who have always, even in the winter, opened them a little should open them a few inches more. Similarly with regard to clothing, suggestive hints are given by horses and cows. Nature, who is a wise and economical tailor, although she does not always get credit for her liberality and skilful fitting, provides the cattle and horses with coats both long and thick at the beginning of winter. These coats in the spring she never fails to remove. But she does not remove them in a day, nor yet in a week or a month. Any groom will tell you that he is covered with hairs for weeks during the spring whenever he gives his horses their morning "wisp." The moral is so obvious that it hardly needs to be drawn. But though it does not need to be drawn, experience shows that it needs to be enforced. Change of clothing should be gradual—very gradual indeed; and fires should be left off gradually—much more gradually than housewives and servants, who hate interference with routine, are disposed to allow of. Everybody should remember that two or three dangerous physiological burglars, to wit, pleurisy, pneumonia, and bronchitis, make their most fruitful harvest in the spring. The best defence against these desperate knaves is painstaking common-sense in the application of simple rules.

But we are assuming that summer is either in full possession of the field or is struggling hard for the mastership. So soon as the victory is gained open-air life in the fullest possible measure should be the rule for all. Why do we never in this country take our meals outside? Even picnics seem to be handed over almost entirely to the *profanum vulgus*, whilst afternoon tea can on no consideration be taken anywhere but in the drawing-room. If it were suggested that *dinner* might be taken on the lawn or in a not too much overlooked back garden, it is to be feared the medical preacher would at once be regarded as hopelessly ignorant of social possibilities and proprieties. In all matters of this kind the "woman is the head of the man," and not the man of the woman as Saint Paul desired. Undoubtedly where woman is the admitted head she rules with a rod of iron. A Bismarck of the sternest type is she, to whom the most stout-hearted of domestic Radicals dare not propose a Reform Bill.

Fresh air and play are the essential conditions of summer pleasure and enrichment in health. Fresh air all will approve, but how can men or women of middle age be expected *to play?* We English are not given to play: we are so solemn, so dignified, so proper, and—shall we say it?—sometimes precise even to priggism. Why should a man or a woman of fifty not play? A very few years ago, at the Edinburgh University games, there was, among other events, a professors' handicap. A lively professor of sixty, mounted on the back of a distinguished baronet of seventy, entered the lists against all comers over a given age, under certain conditions. That particular race was the race of the day. Men and women, assembled in thousands, laughed until they could laugh no more. For one day, at least, the sunshine of youth flooded the whole assembly, and innocent pleasure was completely victorious. Was anybody's dignity impaired by the circumstance? No physic known to man ever produced so large a result of improved health in the world's history as the fine air and healthful sports of the Queen's Park on that memorable occasion.

No doubt the world is full of anxious cares. Undoubtedly to some it is a woeful prison-house, and to all it is a conflict and a strife. But what then? Are we never to banish care and to take up childhood's *rôle* again? Let the nine months of autumn, winter, and spring suffice for stress and strain. In the summer everyone should renew his youth, like the birds and the beasts, the trees and the flowers; and should resolve that by living in the open and giving himself up to the full brightness and joy of the season, he at any rate will cast his cares and his debilities behind him, and do his utmost to give his life and health "a fresh start."

